# The Impact of the *ACTN3* rs1815739 Polymorphism on Physical Performance in Youth Football Players

**DOI:** 10.3390/ijms27219429

**Published:** 2026-10-22

**Authors:** Mesut Cerit, Aysberg Şamil Önlü, Ildus I. Ahmetov, Özlem Özge Yılmaz Hızarcı, Beste Tacal Aslan, Metin Dalip, Korkut Ulucan

**Affiliations:** 1 Department of Exercise and Sports Science, Lokman Hekim University, Ankara 06510, Türkiye; mesut.cerit@lokmanhekim.edu.tr (M.C.); asamil.onlu@lokmanhekim.edu.tr (A.Ş.Ö.); 2 Graduate School of Health Sciences, Ankara University, Ankara 06290, Türkiye; 3 Department of Molecular Biology and Genetics, Lopukhin Federal Research and Clinical Center of Physical Chemical Medicine of Federal Medical Biological Agency, 119435 Moscow, Russia; i.akhmetov@ljmu.ac.uk; 4 Laboratory of Genetics of Aging and Longevity, Kazan State Medical University, 420111 Kazan, Russia; 5 Research Institute for Sport and Exercise Sciences, Liverpool John Moores University, Liverpool L3 5AF, UK; 6 Department of Basic Medical Sciences, Faculty of Dentistry, Marmara University, Istanbul 34854, Türkiye; ozlem.ozge@marmara.edu.tr (Ö.Ö.Y.H.); btacal@gmail.com (B.T.A.); 7 Faculty of Physical Culture and Health, Tetova University, 1220 Tetova, North Macedonia; metin.dalip@unite.edu.mk

**Keywords:** biological maturation, sports genomics, aerobic capacity, countermovement jump, genotype–phenotype association, intermittent recovery

## Abstract

This cross-sectional study examined *ACTN3* rs1815739 (C/T; R577X) genotype distribution and its associations with physical performance and biological maturation in male youth football players. A total of 274 players (11–14 years, *n* = 166; 15–18 years, *n* = 108) from a professional club in North Macedonia completed Yo-Yo Intermittent Recovery, linear sprint, and jump tests, including the drop jump, countermovement jump (CMJ), and squat jump (SJ). Genotype frequencies were compared with the 1000 Genomes European reference population (*n* = 503). *ACTN3* CC (RR) genotype was more frequent in players than in the reference population (48.2% vs. 32.0%; OR = 1.97, 95% CI 1.46–2.67, *p* < 0.0001), as was the C (R) allele (OR = 1.34, 95% CI 1.08–1.66, *p* = 0.009). However, the player genotype distribution deviated from Hardy–Weinberg equilibrium (*p* < 0.001). CMJ height increased by approximately 2.3 cm per one-year increase in maturity offset (*p* < 0.001). In the younger cohort, TT (XX) carriers had lower Yo-Yo distance and estimated VO_2_max than CC and CT (RX) carriers (*p* = 0.007). Exploratory whole-sample models provided limited evidence that the association between maturity offset, and vertical-jump performance (CMJ, SJ) might differ by genotype; however, these interactions were not statistically significant after correction for multiple testing. No significant genotype differences were observed for sprint performance or most other jump-related outcomes. These findings indicate that *ACTN3*–performance associations in youth football players are modest and outcome-specific and should be interpreted cautiously given the external reference population and deviation from Hardy–Weinberg equilibrium.

## 1. Introduction

Athletic performance is a multidimensional phenomenon influenced by the interplay of various factors, including genetic predisposition, environmental conditions, training history, nutrition, and psychological state. Among these, genetic makeup has gained increasing recognition for its role in determining an individual’s training capacity and performance potential [1,2]. Recent advances in molecular genetics, particularly genome-wide association studies, whole-genome sequencing, and whole-exome sequencing, have greatly expanded research into the genetic basis of athletic performance and susceptibility to injuries [3,4,5,6]. These studies indicate that specific genetic variants can significantly influence an individual’s predisposition toward power [7], endurance [8], strength [9], combat [10], and team sports [11]. Within sports genetics, *ACTN3* is one of the most extensively studied genes, with the rs1815739 variant (R577X) receiving particular attention [12].

The *ACTN3* gene encodes the alpha-actinin-3 protein, which is specifically expressed in fast-twitch (type II) muscle fibers and plays a key role in maintaining the structural integrity of the contractile apparatus during muscle contraction [13]. This protein is functionally important for high-intensity activities that require rapid force production, such as sprinting and power-based movements. The most widely studied polymorphism of *ACTN3* is the rs1815739 variant (R577X), a single nucleotide polymorphism characterized by a cytosine (C) to thymine (T) substitution at nucleotide position 1747 in exon 16. This substitution changes an arginine (R) codon to a premature stop codon (X) at amino acid position 577. In individuals homozygous for the X allele (i.e., the TT genotype), functional α-actinin-3 expression is absent, resulting in a nonfunctional alpha-actinin-3 protein, which has been associated with reduced maximal force and power generation [13,14].

Numerous studies have investigated associations between the *ACTN3* rs1815739 polymorphism and anaerobic and aerobic performance [12,15,16]. Carriers of the C allele tend to be over-represented in sports requiring strength and speed, whereas the T allele has been associated with endurance performance [15,17]. Additionally, the C allele has been linked to a greater proportion of fast-twitch muscle fibres [18]. However, associations with individual performance are often weak or inconsistent, and much of the evidence concerns genotype over-representation in athlete populations rather than performance differences within athlete groups. Furthermore, although *ACTN3* rs1815739 is one of the most extensively studied variants in sports genetics, athletic performance is a polygenic trait influenced by numerous genetic variants, each with relatively small individual effects, as well as by environmental and developmental factors.

Adolescence is a critical period for athletic development, characterised by rapid growth, neuromuscular maturation, and changes in performance-related traits. Biological maturation, indexed by maturity offset (years from peak height velocity), can substantially influence physical performance and potentially mask or modify genotype–performance associations [19]. Moreover, the functional effects of the *ACTN3* R577X polymorphism on muscle function and power performance may vary with maturation. Studying youth football players therefore provides an opportunity to investigate whether *ACTN3*–performance associations are evident during adolescence and whether they are independent of, or interact with, biological maturation. This is particularly relevant given the use of talent identification and selection in youth football and the limited evidence supporting the application of genetic information in these contexts.

The present study aimed to evaluate the genotype distribution of the *ACTN3* rs1815739 polymorphism relative to a European reference population, and to investigate its association with physical performance while accounting for biological maturation. We hypothesised that the CC genotype would be over-represented in players relative to the European reference population, and examined whether genotype was associated with individual performance independently of, or in interaction with, maturation.

## 2. Results

### 2.1. Descriptive Characteristics

Descriptive characteristics by age cohort are presented in Table 1.

### 2.2. Genotype Distribution and Case–Control Comparison

The *ACTN3* genotype distribution differed significantly between players and 503 European individuals (χ^2^ = 19.74, *p* < 0.001; Table 2). The odds of carrying the CC genotype were 1.97 times higher in players than in the reference population (48.2% vs. 32.0%; OR = 1.97, 95% CI 1.46–2.67, *p* < 0.0001). Similarly, the odds of carrying the C allele were higher in players than in the reference population (63.5% vs. 56.6%; OR = 1.34, 95% CI 1.08–1.66, *p* = 0.009). When the analysis was restricted to male European individuals (n = 240; CC = 82, CT = 115, TT = 43), the difference in CC genotype frequency remained significant (48.2% vs. 34.2%; OR = 1.79, 95% CI 1.25–2.56, *p* = 0.0013), whereas the difference in C allele frequency was no longer statistically significant (63.5% vs. 58.1%; OR = 1.25, 95% CI 0.975–1.612, *p* = 0.078).

The genotype distribution in the player sample deviated significantly from Hardy–Weinberg equilibrium (Haldane exact test, *p* < 0.001). This deviation was characterized by fewer CT heterozygotes than expected under Hardy–Weinberg equilibrium (84 observed vs. 127 expected; f = 0.34). No significant deviation from Hardy–Weinberg equilibrium was observed in the European reference population.

Because individual ancestry was not documented, the cohort was ethnically heterogeneous, and no regionally matched non-athlete control group was available, this case–control comparison is descriptive and exploratory. The marked heterozygote deficit further limits interpretation, and the higher CC frequency should not be read as evidence of genotype-based selection into football.

### 2.3. Biological Maturation and Performance

Maturity offset was strongly associated with CMJ (β = 2.33 cm per one-year increase in maturity offset, *p* < 0.001; R^2^ = 0.48). Comparing specifications, the chronological-age model fit better than the maturity-offset model (AIC = 1398.6 vs. 1412.4; ΔAIC = 13.8). Because the two predictors are collinear (maturity offset is estimated from chronological age and standing height using the Moore et al. [20] boys equation), genotype associations were consistent across both; maturity-offset models are reported for continuity with the maturation literature, and chronological-age models are provided as sensitivity analyses.

### 2.4. ACTN3 Genotype and Performance by Age Cohort

Genotype comparisons within each age cohort are shown in Table 3. In the 11–14-year cohort, Yo-Yo distance differed by genotype after adjustment for maturity offset (F(2,160) = 5.15, *p* = 0.007, pBH = 0.041, η^2^p = 0.060). Adjusted marginal means were 668 m for CC, 633 m for CT, and 469 m for TT carriers. Tukey-adjusted contrasts showed lower Yo-Yo distance in TT than CC carriers (Δ = −199 m, 95% CI −347 to −51, *p* = 0.005) and in TT than CT carriers (Δ = −163 m, 95% CI −325 to −1, *p* = 0.048); CC and CT did not differ (*p* = 0.798). In the older cohort, no genotype effect remained significant after correction; adjusted CMJ and SJ means were numerically higher in TT carriers, but these differences were non-significant (both pBH = 0.276). No other within-cohort genotype effect remained significant after correction. Estimated VO_2_max was not analysed separately because it is a linear transformation of Yo-Yo distance.

### 2.5. Genotype × Maturation Interactions

**Figure 1:**
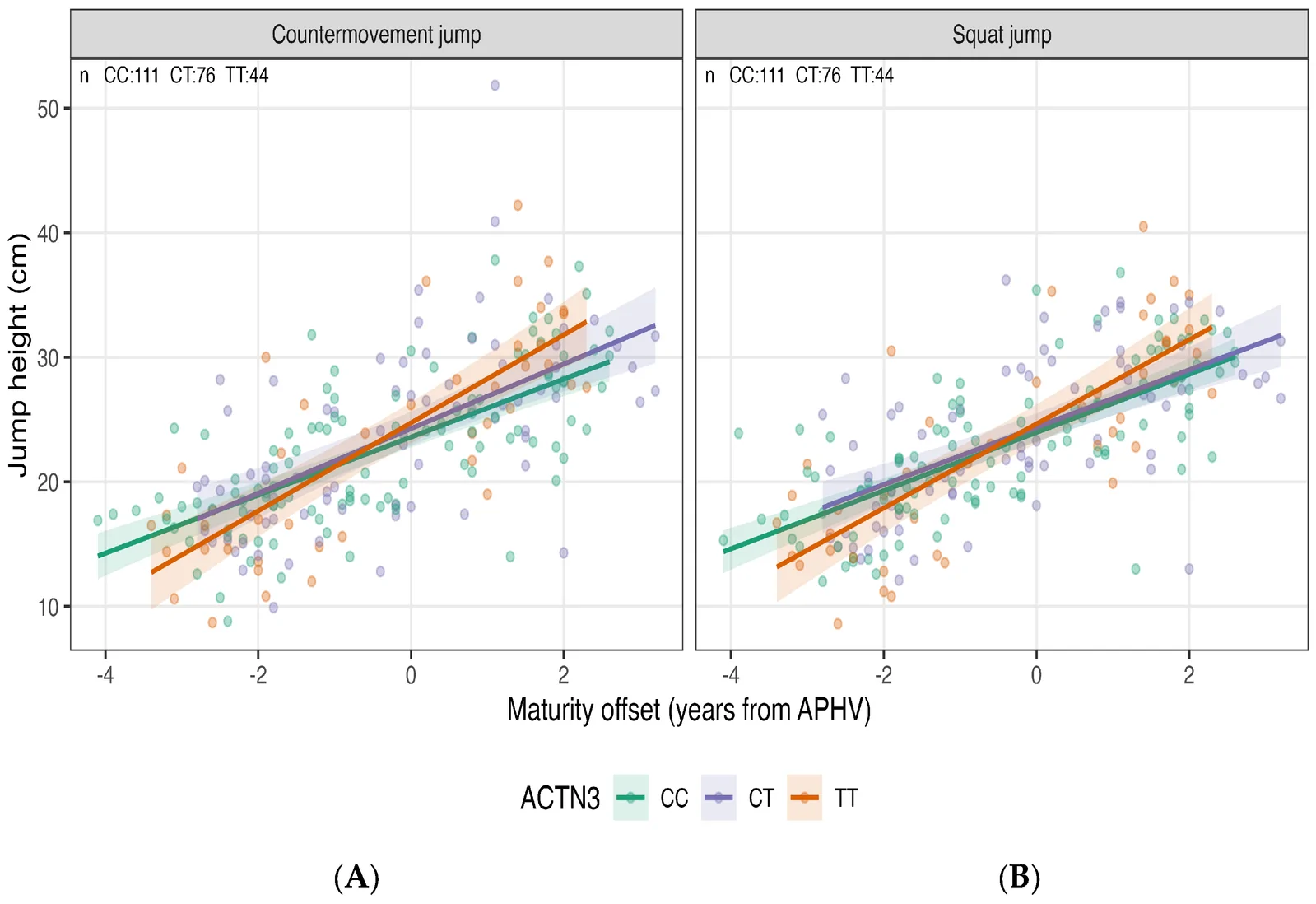
Cross-sectional associations of maturity offset with countermovement jump (CMJ; Panel (**A**)) and squat jump (SJ; Panel (**B**)) height according to ACTN3 genotype. Points represent individual participants, solid lines represent genotype-specific fitted regression lines, and shaded areas represent 95% confidence intervals. Each participant contributed one observation; therefore, the fitted lines represent group-level cross-sectional associations rather than within-participant longitudinal changes.

Whole-sample genotype × maturity-offset interaction models are presented in Table 4. Whole-sample genotype × maturity-offset models are presented in Table 4. None of the five omnibus interactions remained significant after Benjamini–Hochberg correction (all pBH ≥ 0.116). The nominal drift-area interaction (*p* = 0.023) did not survive correction (pBH = 0.116). The CMJ and SJ omnibus interactions were also non-significant (*p* = 0.158 and 0.107, respectively). Genotype-specific fitted regression lines (Figure 1) and maturation slope values according to the genotypes (Table 5) are presented as descriptive and hypothesis-generating only.

## 3. Discussion

This study investigated the distribution of the *ACTN3* rs1815739 polymorphism in youth and adolescent football players (N = 274) and examined its associations with aerobic capacity, sprint performance, vertical jump performance, and biological maturation. The principal findings were a higher frequency of the CC genotype among players than among European individuals, lower Yo-Yo and estimated VO_2_max values in TT carriers within the younger cohort, and exploratory evidence suggesting potentially genotype-dependent maturation slopes for countermovement jump (CMJ) and squat jump (SJ); however, neither the genotype-specific interaction terms nor the omnibus genotype × maturity-offset interactions reached statistical significance after multiple-testing correction. No significant genotype differences were observed for sprint performance, and most other performance outcomes did not differ by genotype.

The CC genotype and C allele were the most frequent genotype and allele, respectively, in the player sample. This distribution is consistent with previous reports of relatively high C-allele frequencies among football players and athletes participating in power-oriented sports [21,22,23,24,25]. Compared with the European reference population, the odds of carrying the CC genotype were 1.97 times higher among the players. This finding is compatible with previous evidence linking the C allele, corresponding to functional α-actinin-3 expression, with strength- and power-related athletic phenotypes [16,26,27,28]. However, the observed case–control association does not demonstrate a causal effect of the genotype on participation or selection into football.

Previous studies have associated functional α-actinin-3 expression with type II muscle-fibre characteristics, muscle strength, explosive performance, and recovery following high-intensity exercise [19,29,30]. Nevertheless, the present findings did not demonstrate a consistent C-allele advantage across the measured sprint and jump outcomes. Genotype differences were not significant for sprint performance in either cohort, and no significant within-cohort genotype differences were detected for CMJ, SJ, drop jump, or reactive strength index. Thus, the higher CC frequency observed in the players was not accompanied by uniformly superior physical performance among CC carriers.

In the 11–14-year cohort, TT carriers had lower Yo-Yo distance and estimated VO_2_max values than CC and CT carriers. This result contrasts with the classical physiological model in which α-actinin-3 deficiency is associated with greater oxidative capacity and a higher proportion of type I muscle fibres [13,31,32]. The Yo-Yo IR1 assesses repeated high-intensity running with brief recovery periods and is influenced by several physiological and sport-specific factors rather than oxidative capacity alone. Accordingly, the expected endurance-related characteristics of the TT genotype may not translate directly into superior intermittent-running performance. Because the TT subgroup contributing to these outcomes was relatively small (n = 34), this finding should be interpreted cautiously and examined in independent samples.

Exploratory whole-sample models provided limited evidence that the association between maturity offset and vertical-jump performance (CMJ, SJ) might differ by genotype, with descriptively steeper maturation slopes in TT carriers; however, the omnibus interactions were not statistically significant after correction for multiple testing and should be regarded as hypothesis-generating. Because the study was cross-sectional, these slopes cannot be interpreted as longitudinal gains or developmental catch-up. No significant genotype × maturity-offset interaction was observed for drop jump, reactive strength index, or drift area, suggesting that the interaction was restricted to the vertical jump outcomes.

Although the TT genotype has traditionally been associated with a more oxidative muscle phenotype, this physiological characteristic does not necessarily confer superior performance in the Yo-Yo IR1, particularly during early adolescence. Children rely relatively more on oxidative metabolism and possess a less developed glycolytic capacity than mature individuals; therefore, the lower Yo-Yo performance observed in TT carriers cannot be attributed to a general predominance of anaerobic metabolism before maturation [33]. Rather, the Yo-Yo IR1 combines aerobic energy provision and rapid recovery with repeated accelerations, decelerations, and high-intensity running bouts [34,35]. Functional α-actinin-3 expression in CC and CT carriers may therefore support the neuromuscular and repeated high-intensity components of the test, whereas the oxidative characteristics associated with the TT genotype may not provide the same advantage in this football-specific context. Related findings have been reported in Chinese football players aged 13–15 years, among whom RR carriers demonstrated superior speed and explosive performance without an aerobic advantage for XX carriers [28]. Furthermore, lower match-running performance has been reported in professional football players with the XX genotype [17], while recent meta-analytic evidence indicates that the proposed association between the XX genotype and superior endurance performance is inconsistent [12]. The present findings may therefore reflect maturation-dependent expression of the *ACTN3* phenotype or differences in responsiveness to football-specific demands; however, because the current data are cross-sectional, this adaptability explanation should be regarded as a hypothesis requiring longitudinal confirmation.

Sprint performance did not differ significantly among the genotype groups in either age cohort. Although the C allele has previously been associated with sprint- and power-related performance [16,26,28,36,37], the present findings did not provide evidence of a genotype effect on sprint time. Sprint and football performance are multifactorial characteristics influenced by training status, technique, neuromuscular coordination, biological maturation, positional demands, and environmental factors [38,39]. The influence of a single polymorphism is therefore likely to be small relative to these combined determinants.

### Limitations

Several limitations should be considered. First, the cross-sectional design precludes causal or longitudinal interpretations of the genotype × maturity-offset associations. Second, participants were male players from a single professional football club and geographic setting, which may limit generalizability. Third, sample size varied across outcomes because of missing measurements, and the TT subgroup was comparatively small in several analyses. Fourth, the two age cohorts completed different Yo-Yo test levels, preventing a direct whole-sample analysis of Yo-Yo distance and estimated VO_2_max. Fifth, the genotype distribution deviated markedly from Hardy–Weinberg equilibrium (*p* < 0.001), with fewer CT heterozygotes than expected (84 vs. 127; f = 0.34). Population substructure (Wahlund effect), non-random sampling, and genotyping error may have contributed to this deviation. Although duplicate genotyping showed 100% concordance, only 20 samples were reassessed, preventing the definitive exclusion of genotyping error. In addition, individual ancestry was not documented, and the EUR reference population from the 1000 Genomes Project was not regionally matched to the study cohort. These factors may have influenced the observed genotype distributions and estimated odds ratios, warranting caution when interpreting the comparison with the reference population. Consequently, the findings do not establish an association between *ACTN3* genotype and selection into football. Finally, only one genetic polymorphism was examined, whereas football participation and performance are polygenic and are likely influenced by numerous genetic variants and their interactions with environmental factors [40,41]. Future studies should incorporate polygenic scores and additional candidate variants to provide a more comprehensive picture of the genetic architecture of youth football performance.

## 4. Materials and Methods

### 4.1. Participants and Ethical Considerations

The study was conducted in accordance with the Declaration of Helsinki, and ethical approval was granted by the Lokman Hekim University Non-Interventional Clinical Research Ethics Committee (decision number: 2025-105; date of approval: 28 April 2025).

This study involved 274 male youth football players aged 11–14 years (youth category; n = 166) and 15–18 years (adolescent category; n = 108), all registered with a professional football club in North Macedonia. Individual nationality and ancestry were not systematically recorded; the cohort was predominantly of Southeastern European (Balkan) background but was not ethnically homogeneous, as some players originated from outside the region. All athletes regularly engaged in structured training programs including technical, tactical, and conditioning exercises (focused on speed, strength, and endurance) six days per week, with each session lasting approximately 45 to 75 min. Players had between 2 and 10 years of structured football training experience. To be eligible for the study, players were required to be registered members of the club, to participate regularly in the club’s structured training program, and to be able to complete the physical testing procedures safely. Players were excluded if they had a current musculoskeletal injury, a medical or health condition that contraindicated maximal physical exercise or could place their health at risk, or were unable to complete the prescribed testing protocol. Before participation, written informed consent was obtained from players aged 18 years. For participants younger than 18 years, written informed consent was obtained from a parent or legal guardian, and written assent was obtained from the player. Permission to conduct the study was also obtained from the football club.

### 4.2. Assessment of Biological Maturation

Standing height was measured to the nearest 0.1 cm with a calibrated stadiometer (Seca 213, Seca GmbH, Hamburg, Germany); body mass and body composition were measured to the nearest 0.1 kg by bioelectrical impedance analysis (Avis333), with participants unshod and in light clothing. Chronological age was computed in decimal years from date of birth and assessment date. Biological maturation was estimated as the maturity offset (years from age at peak height velocity) using the sex-specific equation of Moore et al. [20] for boys, which requires only chronological age and standing height: maturity offset = −7.999994 + 0.0036124 × (age × standing height). Sitting height was not measured; the Moore et al. [20] age-and-height equation was therefore used rather than the Mirwald et al. [42] equation, which requires sitting height. Predicted APHV = chronological age − maturity offset [42].

### 4.3. External European Reference Population

In addition, the *ACTN3* genotype distribution and allele frequencies of the youth football players were compared with data from 503 individuals (240 males and 263 females) in the European population (EUR) of the 1000 Genomes Project (Phase 3), accessed through Ensembl on 30 August 2025. The reference samples were obtained from adult donors aged over 18 years who reported being healthy at the time of sample collection. Individual age data and information on physical fitness, training history, and athletic participation were unavailable. The EUR dataset was selected as a broad European reference because a regionally matched non-athlete control group was unavailable. However, it does not specifically reflect the ancestry composition of the present Balkan-based cohort. Accordingly, the comparison was considered descriptive and exploratory rather than a matched case–control analysis. The participants in the present study were drawn from the same cohort as those in a companion study examining single-leg reactive strength and dynamic stability, which has not yet been published. The two studies address distinct research questions and involve no overlapping analyses.

### 4.4. Exercise and Performance Measurement Methods

All participating athletes were already familiar with the testing procedures through their regular training and evaluation routines. Before the tests, each player completed general and sport-specific warm-up exercises, followed by a three- to five-minute rest to ensure optimal recovery. All performance tests were conducted on separate, non-consecutive days, and each test was administered according to its own standardized protocol. The tests were performed indoors under comparable environmental conditions. High-intensity intermittent running and recovery capacity were assessed using the Yo-Yo Intermittent Recovery Tests (Yo-Yo IRT Levels 1 and 2), administered indoors and guided by standardized audio signals. Sprint performance over 20 and 30 metres was recorded using the Witty Speed (Microgate) electronic timing system. The squat jump (SJ) was used to assess concentric lower-body jump performance, the countermovement jump (CMJ) to assess explosive lower-body performance involving the stretch-shortening cycle, and the drop jump (DJ) to assess reactive strength and fast stretch-shortening-cycle performance.

### 4.5. Yo-Yo Intermittent Recovery Tests (Yo-Yo IRT 1 and 2)

The Yo-Yo IRT Levels 1 and 2 were conducted to assess high-intensity intermittent running and recovery capacity. Players in the younger cohort completed the Yo-Yo IRT Level 1, whereas players in the older cohort completed the Yo-Yo IRT Level 2. These tests consist of repeated 20-metre shuttle runs at progressively increasing speeds, interspersed with 10 s active recovery intervals, continued until the participant twice failed to reach the finish line within the required time or voluntarily stopped because of exhaustion; the cumulative distance covered was recorded for each player. To minimise environmental variability, all trials were conducted indoors [34,43,44]. Distances were converted to estimated VO_2_max using established equations [34,35]: for Yo-Yo IRT Level 1, VO_2_max = (distance in metres × 0.0084) + 36.4; for Yo-Yo IRT Level 2, VO_2_max = (distance in metres × 0.0136) + 45.3. Accordingly, the calculated VO_2_max values were treated as estimates rather than as direct measurements of maximal oxygen uptake.

### 4.6. Sprint Tests (20 m and 30 m)

Linear sprint performance was assessed using two age-appropriate distances. Participants in the younger cohort (11–14 years) completed a 20-metre sprint, while those in the older cohort (15–18 years) performed a 30-metre sprint. Each sprint began from a stationary standing position, with times recorded using a photocell timing system (Witty Speed, Microgate). Sprint performance was segmented into initial acceleration (0–5 m), transition (5–10 m), and maximal speed (10 m to finish). Participants completed two attempts with a five-minute rest, and the fastest time was used for analysis. All sessions were conducted indoors under consistent conditions.

Because the two cohorts performed different sprint distances (20 m for 11–14 years, 30 m for 15–18 years), sprint times were compared between genotypes within each age cohort only, using total sprint time; cross-cohort sprint comparisons were not performed.

### 4.7. Jump Tests

Vertical jump ability was assessed using DJ, CMJ, and SJ tests on an infrared contact platform (Optojump; Microgate, Bolzano, Italy). For the SJ, participants assumed a static position with approximately 90° of knee flexion, maintained this position for approximately 2 s, and then jumped vertically as high as possible without performing a preliminary countermovement. Trials involving a countermovement were considered invalid and repeated. For the CMJ, participants began from an upright standing position, performed a rapid downward movement to a self-selected depth, and immediately followed this movement with maximal vertical propulsion. For the DJ, participants stepped forward from a 30 cm box without jumping upward, landed with both feet on the measurement area, and were instructed to minimize ground-contact time while jumping vertically as high as possible immediately after landing. All jumps were performed with the hands on the hips, executed three times with a 15 s interval, and the best score was recorded. Flight time (t) was used to compute jump height (h = gt^2^/8, g = 9.81 m/s^2^) [45]. For the drop jump, the OptoJump device generated data corresponding to the descent, landing, and subsequent rebound phases. The device-derived output was manually reviewed to verify that these phases had been correctly identified and that the rebound-jump value had been correctly recorded. Drop-jump heights below 8 cm were classified as unsuccessful attempts in accordance with the device protocol. Trials for which valid data could not be obtained were excluded. Each participant completed three trials, and the best valid rebound-jump result was retained for analysis.

### 4.8. Single-Leg Drift Protocol (Reactive Strength and Dynamic Stability)

Reactive strength and dynamic stability were assessed using the standardized Drift protocol application of the OptoJump Next system (Microgate, Bolzano, Italy). The protocol was administered using the standardized settings and instructions provided by the device application. Participants performed five consecutive single-leg jumps under four conditions: right-leg anterior–posterior drift, right-leg medio-lateral drift, left-leg anterior–posterior drift, and left-leg medio-lateral drift. Reactive strength index (RSI) was calculated as jump height (m) divided by ground contact time (s). Dynamic stability was quantified using the drift-area output generated by the OptoJump Drift application from the device-derived landing-position data. A larger drift area indicated poorer dynamic stability. RSI and drift area were averaged across the four conditions to provide one value per participant.

The same cohort and single-leg measurements were used in a companion study examining age-, leg-, and plane-related effects. The present study addressed a distinct genetic question by examining *ACTN3* genotype and genotype × maturation associations.

### 4.9. Genetic Analysis

For genetic analyses, oral buccal swab samples were collected from each participant using sterile cotton swabs under standardized procedures and stored in phosphate-buffered saline (PBS) until processing. Genomic DNA isolation was performed using a commercial kit (PureLink™ Genomic DNA Mini Kit, Thermo Fisher Scientific, Carlsbad, CA. USA) according to the manufacturer’s instructions. The concentration of the obtained DNA samples was accurately measured via fluorescence-based quantification using a Qubit 4 Fluorometer (Thermo Fisher Scientific, USA) with the appropriate assay kit. The isolated DNA samples were stored at −20 °C for subsequent analyses. Genotyping of the *ACTN3* rs1815739 polymorphism was performed by real-time PCR using an Applied Biosystems real-time PCR system and FAM- and VIC-labelled fluorescent probes (Assay ID: C____590093_1_). Each reaction had a total volume of 10 µL and contained 5 µL of real-time PCR master mix, 3.5 µL of sterile H_2_O, 0.5 µL of assay mix, and 1 µL (10 ng) of genomic DNA. Reactions were prepared in a 0.1 mL plate and processed in the real-time PCR system. Duplicate genotyping was performed for quality-control purposes on a randomly selected subset of 20 samples (7.3% of the player cohort), with 100% concordance. However, this limited assessment cannot exclude genotyping errors across the entire cohort, particularly systematic misclassification.

### 4.10. Statistical Analyses

Analyses were performed in R (version 4.4.2; R Core Team, 2024). Descriptive statistics are presented as mean ± standard deviation for continuous variables and as frequencies (n, %) for categorical variables.

Genotype and allele distributions of the players were compared with 1000 Genomes European population data using Pearson chi-square tests, with odds ratios (OR) and 95% confidence intervals calculated for the CC genotype and the C allele; this case–control comparison was treated as descriptive and exploratory. Hardy–Weinberg equilibrium (HWE) was assessed using the Haldane exact test, and the inbreeding coefficient (f) was calculated. HWE is the expected distribution of genotypes in a population when mating is random, and no forces such as selection, migration, or genetic drift are acting. Under HWE, genotype frequencies can be predicted from allele frequencies using the equation p^2^ + 2pq + q^2^ = 1, where p and q represent the frequencies of the two alleles. Deviation from HWE may indicate genotyping error, population substructure (e.g., the Wahlund effect, where a sample combines genetically distinct subgroups), non-random mating, or selection (e.g., in sport). For the case–control comparison, two types of odds ratios were calculated to distinguish between allele-level and genotype-level associations. First, allele-level ORs were computed by comparing the frequency of the C allele to the T allele between players and the European reference population, using a 2 × 2 contingency table (C vs. T alleles). Second, genotype-level ORs were computed for the CC genotype versus the combined non-CC genotypes (CT + TT), again using a 2 × 2 contingency table. This approach was chosen because the CC genotype represents homozygosity for the functional C allele, whereas the CT and TT genotypes do not. The OR for the CC genotype therefore reflects the odds of being homozygous for the C allele relative to not being homozygous, rather than the odds of carrying at least one C allele. A separate C-carrier analysis (CC + CT vs. TT) was also performed and is reported in the text for completeness. All ORs are reported with 95% confidence intervals, and the corresponding odds values can be reconstructed from the genotype and allele frequencies presented in Table 2. This case–control comparison was treated as descriptive and exploratory throughout.

Biological maturation was operationalised as the maturity offset (years from age at peak height velocity), estimated with the Moore et al. [20] equation for boys. To characterise the maturation–performance relationship, two alternative linear models were fitted for each jump outcome: one using chronological age as the predictor and one using maturity offset as the predictor. Both models included the same outcome variable and were fitted to the same sample. The Akaike Information Criterion (AIC) was then computed for each model using the AIC() function in R. The AIC is a measure of relative model quality that balances goodness of fit against model complexity, with lower values indicating a better trade-off between fit and parsimony. The model with the lower AIC was considered to provide a better fit to the data. Because maturity offset is estimated from chronological age and standing height, the two predictors are collinear; the AIC comparison was therefore used descriptively to characterise which predictor better captured the maturation–performance relationship, rather than to select a single ‘true’ model. Genotype associations were consistent across both specifications, and maturity-offset models are reported for continuity with the maturation literature, with chronological-age models provided as sensitivity analyses.

Within each age cohort, genotype effects on each performance outcome were tested using analysis of covariance (ANCOVA) with genotype as a fixed factor and maturity offset as a covariate (reference genotype = CC), rather than unadjusted one-way ANOVA, so that genotype associations were estimated independently of maturation. Estimated marginal means and Tukey-adjusted pairwise contrasts (with 95% confidence intervals) were derived from these models, and partial eta-squared (η^2^p) was reported as the effect size. Because estimated VO_2_max is a linear transformation of Yo-Yo distance, only Yo-Yo distance was analysed. Yo-Yo comparisons were conducted within cohort, as the two cohorts completed different test levels (IR1 for 11–14 years, IR2 for 15–18 years).

Genotype × maturation interactions were examined across the whole sample using linear models that included genotype, maturity offset, and their interaction (reference genotype = CC). For each outcome, the interaction was evaluated with an omnibus test of the joint genotype × maturity-offset terms (CT and TT); genotype-specific maturation slopes were estimated as secondary, descriptive quantities.

Multiple comparisons were controlled with the Benjamini–Hochberg (BH) false-discovery-rate procedure applied within pre-defined families: the within-cohort genotype comparisons formed one family, and the omnibus genotype × maturation interaction tests across outcomes formed a separate family. Both raw and BH-adjusted *p*-values are reported. Interaction results that did not survive BH correction are reported as exploratory. Owing to missing measurements, sample sizes varied across outcomes; available-case analysis was used without listwise deletion. Statistical significance was set at α = 0.05 (two-tailed).

## 5. Conclusions

In conclusion, the CC genotype was more frequent among youth football players than in the European reference population. Within the younger cohort, TT carriers had lower Yo-Yo and estimated VO_2_max values than CC and CT carriers, whereas exploratory genotype × maturation interactions for CMJ and SJ did not survive correction for multiple testing. No consistent genotype differences were identified for sprint performance or most jump-related outcomes. These findings indicate that associations between *ACTN3* rs1815739 and physical performance in youth football players are limited and outcome-specific, and that performance during this period is dominated by biological maturation. Genetic information should therefore not be used in isolation for talent identification, athlete selection, or training prescription. Future research should adopt a polygenic approach that accounts for multiple genetic variants and their interactions with maturation and training.

### Practical Applications

Because individual performance was only weakly related to genotype, *ACTN3* testing cannot currently justify genotype-based training prescription or athlete selection in youth football. Any use of genetic information should be one minor, non-deterministic input within a comprehensive, maturation-aware evaluation framework.

## Figures and Tables

**Table 1:** Descriptive characteristics and performance by age cohort (mean ± SD).

Variable	11–14 Years (n = 166)	15–18 Years (n = 108)
CC, n (%)	81 (48.8)	51 (47.2)
CT, n (%)	51 (30.7)	33 (30.6)
TT, n (%)	34 (20.5)	24 (22.2)
Height (cm)	160.6 ± 11.6	175.9 ± 5.5
Body mass (kg)	49.3 ± 12.3	65.9 ± 8.0
Drop jump (cm)	21.3 ± 5.7	29.9 ± 5.3
CMJ (cm)	19.8 ± 5.1	29.4 ± 5.4
Squat jump (cm)	20.4 ± 5.1	29.0 ± 4.4
VO_2_max (mL·kg^−1^·min^−1^)	41.6 ± 2.9	53.0 ± 3.1
Sprint time (s)	3.87 ± 0.49 (20 m)	4.30 ± 0.33 (30 m)

Values are mean ± SD; valid n varies by measure. CMJ = countermovement jump. Estimated VO_2_max is a linear transformation of Yo-Yo distance (IR1 for 11–14, IR2 for 15–18), not an independent measurement. Sprint distances differed by cohort (20 m vs. 30 m) and are not comparable across groups.

**Table 2:** *ACTN3* genotype and allele distribution in players versus 1000 Genomes European individuals.

	Players (n = 274)	EUR (n = 503)	OR (95% CI)
CC, n (%)	132 (48.2)	161 (32.0)	1.97 (1.46–2.67)
CT, n (%)	84 (30.7)	247 (49.1)	—
TT, n (%)	58 (21.2)	95 (18.9)	—
C allele (%)	63.5	56.6	1.34 (1.08–1.66)
T allele (%)	36.5	43.4	—
HWE *p*-value	<0.001	0.988	—
Inbreeding coefficient (f)	0.34	0.00	—

1000 Genomes Project Phase 3, European (EUR) super-population (accessed via Ensembl). OR = odds for the CC genotype/C allele in players relative to reference population. HWE = Hardy–Weinberg equilibrium, assessed using the Haldane exact test in both groups; f = inbreeding coefficient.

**Table 3:** Maturity-adjusted performance by *ACTN3* genotype within each age cohort.

Variable	CC EMM (95% CI), n	CT EMM (95% CI), n	TT EMM (95% CI), n	F (df)	*p*	pBH	η^2^p
***11–14 years***							
CMJ (cm)	19.97 (19.00, 20.95), 77	20.19 (18.94, 21.44), 47	18.90 (17.31, 20.50), 29	0.86 (2,149)	0.427	0.640	0.011
SJ (cm)	20.38 (19.45, 21.30), 77	20.98 (19.80, 22.17), 47	19.30 (17.79, 20.81), 29	1.49 (2,149)	0.228	0.567	0.020
DJ (cm)	21.13 (20.01, 22.26), 69	22.14 (20.75, 23.54), 45	20.37 (18.56, 22.17), 27	1.27 (2,137)	0.284	0.567	0.018
RSI (m/s)	1.66 (1.61, 1.71), 77	1.66 (1.60, 1.71), 48	1.70 (1.62, 1.77), 31	0.45 (2,152)	0.637	0.761	0.006
Yo-Yo (m)	668 (601, 735), 81	633 (546, 719), 49	469 (366, 573), 34	5.15 (2,160)	0.007	0.041	0.060
Sprint (s)	3.84 (3.74, 3.95), 75	3.91 (3.77, 4.05), 46	3.86 (3.69, 4.04), 30	0.27 (2,147)	0.761	0.761	0.004
***15–18 years***							
CMJ (cm)	28.39 (26.54, 30.24), 34	29.24 (27.23, 31.25), 29	32.13 (29.33, 34.93), 15	2.50 (2,74)	0.089	0.276	0.063
SJ (cm)	28.70 (27.18, 30.22), 34	28.36 (26.70, 30.01), 29	31.37 (29.07, 33.67), 15	2.47 (2,74)	0.092	0.276	0.062
DJ (cm)	29.85 (28.05, 31.65), 34	29.57 (27.58, 31.56), 28	31.36 (28.64, 34.08), 15	0.60 (2,73)	0.553	0.749	0.016
RSI (m/s)	1.50 (1.44, 1.55), 35	1.48 (1.42, 1.54), 29	1.49 (1.41, 1.57), 16	0.12 (2,76)	0.883	0.883	0.003
Yo-Yo (m)	577 (504, 649), 42	593 (510, 676), 32	526 (415, 636), 18	0.47 (2,88)	0.625	0.749	0.011
Sprint (s)	4.19 (4.09, 4.30), 40	4.32 (4.21, 4.44), 31	4.33 (4.17, 4.48), 17	1.83 (2,84)	0.167	0.335	0.042

Values are estimated marginal means (95% CI) adjusted for Moore maturity offset, with valid n reported for each genotype group. F tests are from ANCOVA models including genotype and maturity offset. η^2^p = partial eta-squared. The younger cohort completed Yo-Yo IR1 and the older cohort completed Yo-Yo IR2; therefore, Yo-Yo comparisons are restricted to within-cohort analyses. Missing measurements were handled using available-case analysis.

**Table 4:** Genotype × maturation linear models: coefficients, 95% CI, per-term *p*-values, and outcome-level omnibus interaction tests.

Outcome	Term	β	95% CI	*p*	Omnibus Interaction *p* (BH)
*CMJ height* (*n* = 231, *R*^2^ = 0.511)					
	Intercept	21.153	20.19, 22.12	<0.001	*p* = 0.158 (pBH = 0.264)
	genotype CT	0.391	−1.18, 1.96	0.625	
	genotype TT	0.011	−1.79, 1.81	0.991	
	maturity	2.591	2.03, 3.15	<0.001	
	CT × maturity	0.287	−0.60, 1.18	0.526	
	TT × maturity	0.926	−0.02, 1.87	0.055	
*SJ height* (*n* = 231, *R*^2^ = 0.547)					
	Intercept	21.516	20.66, 22.37	<0.001	*p* = 0.107 (pBH = 0.264)
	genotype CT	0.494	−0.90, 1.89	0.486	
	genotype TT	−0.300	−1.89, 1.29	0.711	
	maturity	2.610	2.12, 3.11	<0.001	
	CT × maturity	−0.116	−0.91, 0.67	0.772	
	TT × maturity	0.787	−0.05, 1.63	0.066	
*DJ height* (*n* = 218, *R*^2^ = 0.484)					
	Intercept	22.201	21.16, 23.24	<0.001	*p* = 0.636 (pBH = 0.795)
	genotype CT	0.868	−0.80, 2.54	0.306	
	genotype TT	−0.172	−2.09, 1.75	0.860	
	maturity	2.847	2.26, 3.43	<0.001	
	CT × maturity	−0.293	−1.22, 0.64	0.535	
	TT × maturity	0.224	−0.78, 1.23	0.659	
*RSI* (*n* = 236, *R*^2^ = 0.260)					
	Intercept	1.646	1.61, 1.68	<0.001	*p* = 0.867 (pBH = 0.867)
	genotype CT	−0.008	−0.07, 0.05	0.798	
	genotype TT	0.023	−0.05, 0.09	0.513	
	maturity	−0.066	−0.09, −0.04	<0.001	
	CT × maturity	−0.005	−0.04, 0.03	0.784	
	TT × maturity	0.006	−0.03, 0.04	0.745	
*Drift area (log)* (*n* = 236, *R*^2^ = 0.342)					
	Intercept	0.932	0.85, 1.02	<0.001	*p* = 0.023 (pBH = 0.116)
	genotype CT	−0.081	−0.22, 0.06	0.260	
	genotype TT	−0.186	−0.35, −0.03	0.022	
	maturity	0.134	0.08, 0.18	<0.001	
	CT × maturity	0.055	−0.02, 0.014	0.174	
	TT × maturity	0.117	0.03, 0.20	0.007	

β = unstandardised coefficient; CC is the reference genotype; maturity = Moore maturity offset. The omnibus interaction *p*-value jointly tests the CT × maturity and TT × maturity terms. pBH values were adjusted across the five omnibus interaction tests. Drift area was natural-log transformed. No omnibus interaction remained significant after correction. Intercepts represent the predicted outcome at maturity offset = 0 (APHV) for the CC genotype and therefore differ from the cohort-specific values in Table 1 and Table 3. Individual observations and genotype-specific fitted regression lines for the cross-sectional associations of maturity offset with CMJ and SJ height are presented in Figure 1.

**Table 5:** Maturation slope (cm per one-year increase in maturity offset) within each genotype for CMJ and squat jump.

Outcome	CC Slope	CT Slope	TT Slope
CMJ (cm per one-year maturity offset)	2.59 (0.28)	2.88 (0.35)	3.52 (0.39)
SJ (cm per one-year maturity offset)	2.61 (0.25)	2.49 (0.31)	3.40 (0.34)

Values are slope (SE). The corresponding omnibus interactions were not significant after multiplicity correction; therefore, these slopes are descriptive and exploratory.

## Data Availability

The data supporting the findings of this study are available from the corresponding author upon reasonable request.
